# Obesity and Other Nutrition Related Abnormalities in Pre-Dialysis Chronic Kidney Disease (CKD) Participants

**DOI:** 10.3390/nu12123608

**Published:** 2020-11-24

**Authors:** Zarina Ebrahim, M. Rafique Moosa, Renée Blaauw

**Affiliations:** 1 Division of Human Nutrition, Stellenbosch University, Cape Town 8000, South Africa; rb@sun.ac.za; 2 Department of Medicine, Stellenbosch University, Cape Town 8000, South Africa; rmm@sun.ac.za

**Keywords:** pre-dialysis nutrition assessment, nutrition assessment in chronic kidney disease, obesity in chronic kidney disease

## Abstract

Chronic kidney disease (CKD) is increasing in sub-Saharan Africa. Undernutrition has been prevalent amongst end stage CKD patients, with limited data on the prevalence of obesity. The aim of this study was to assess the nutritional status of CKD patients using various methods sensitive to over and under-nutrition. Stage 3 to 5 CKD patients (glomerular filtration rate (GFR) < 60 mL/min/1.73 m^2^) attending a pre-dialysis clinic in Cape Town, were enrolled. Exclusion criteria included infectious and autoimmune conditions. Sociodemographic, clinical and biochemical data were collected, and anthropometric measurements were performed. Dietary intake was measured with a quantified food frequency questionnaire (FFQ). Statistical Package for the Social Sciences (SPSS) version 26 was used for statistical analysis. Seventy participants, with mean age of 41.8 ± 11.8 years, 52.9% females and 47.1% males were enrolled. Participants enrolled mainly had stage 5 kidney failure. Thirty percent were overweight (21) and 25 (36%) were obese, 22 (60%) of females were overweight and obese, while 13 (39.4%) of males were predominantly normal weight. Abdominal obesity was found in 42 (60%) of participants, mainly in females. Undernutrition prevalence was low at 3%. Dietary assessment showed a high sugar and protein intake. There was a high prevalence of overweight, obesity and abdominal obesity in CKD stage 35 patients, with unhealthy dietary intake and other nutritional abnormalities.

## 1. Introduction

Malnutrition in the form of overweight and obesity has been increasing in prevalence in chronic kidney disease (CKD) [1,2]. This is likely due to the increase in chronic diseases of lifestyle, since many are risk factors for the development of CKD [2,3]. Obesity is closely related to cardiovascular and other metabolic diseases such as insulin resistance, type 2 diabetes mellitus and chronic inflammation through various mechanisms [3,4]. Obesity also induces glomerular hyperfiltration [3]. An increased body mass index (BMI) above 25 kg/m^2^ has recently been associated with a progressively increased risk of CKD stages 4 to 5 [5]. It is therefore important to identify and treat obesity in early CKD to prevent further deterioration of the disease.

CKD results in many nutritional status abnormalities as glomerular filtration rate decreases. These include bone mineral abnormalities, anemia, inflammation, electrolyte imbalances, undernutrition, increased catabolism and appetite problems [6,7]. This results in many dietary restrictions. Due to the restrictive nature of the diet and due to symptoms of the disease, diet adherence is low [8]. These factors in addition to obesity will negatively influence the nutritional status of CKD patients.

The International Society of Renal Nutrition and Metabolism (ISRNM) developed a set of criteria focusing on body weight, muscle mass, biochemical assessment and dietary intake to diagnose undernutrition, specifically in CKD patients. The Subjective Global (SGA) assessment tool has also been used to diagnose undernutrition in CKD; however due to its subjectivity, the ISRNM suggest that it not be used to diagnose undernutrition, but should rather be used as a clinical marker [9,10]. Neither of these have criteria for identifying overweight or obesity as a malnutrition risk.

The main focus of research has been on the high prevalence of malnutrition in terms of protein energy undernutrition in pre-dialysis CKD patients [6,11,12]. The ISRNM criteria are used to diagnose undernutrition in some of the studies [9]. The KoreaN cohort study for Outcome in patients With Chronic Kidney Disease (KNOW-CKD) study reported a prevalence of 9%, while a Nigerian study reported a prevalence of undernutrition of 47% using the ISRNM criteria, however the two studies used the criteria in different ways [13,14]. No information was provided for the rest of the BMI categories for either studies, although the average BMI approached the overweight category. Studies using the SGA has also found high rates of undernutrition [1,10,15]. These studies demonstrate that different screening criteria are used to diagnose undernutrition prevalence and that there is a failure to report on the extent of overweight or obesity.

It is possible that less emphasis is placed on obesity because of the obesity paradox; this refers to the reduced mortality outcomes that has been shown in CKD with higher BMI’s [16]; however there are conflicting results particularly within the pre-dialysis patient group. Herrington et al. [5] suggests reasons why the obesity paradox theory may not be substantiated in CKD; these include a low BMI due to individuals being in an overall poorer health condition and often in a cachectic state, they also suggest methodological and enrolment flaws in some of the studies reporting these associations.

This study assessed the nutritional status of CKD pre-dialysis participants using different measures that may be sensitive to both over and undernutrition.

## 2. Materials and Methods

This cross-sectional study investigated the nutritional status of 70 stage 3 to 5 CKD participants attending the pre-dialysis clinic at Tygerberg Hospital in Cape Town, South Africa, between 1 August 2018 and 30 September 2019. Participants files were screened and were selected if their GFR’s were less than 60 mL/min/1.73 m^2^ at their routine Renal Clinic visit and they were older than 18 years, provided exclusion criteria did not apply. The latter included any infectious diseases that may affect nutritional status, immunological conditions, severe gastrointestinal disease, pregnancy, diabetes and participants who were expected to start dialysis in the next 2 months. This study represents the baseline data of a randomized control study investigating uraemic toxins, gut microbiome and CKD outcomes using a prebiotic supplement. A total of 70 participants were enrolled for the study and the baseline data is presented here.

Ethics approval was obtained (S18/03/064) from Stellenbosch University Health Research Ethics Committee.

Data collected included socio-demographic and relevant biochemical and clinical information.

The following anthropometric measurements were performed according to World Health Organization (WHO) standards [17]: weight (kg), height (cm), waist circumference (cm), mid-upper arm circumference (MUAC) (cm) and triceps (mm) [18] and reported by group and gender. Appendix A indicates the formulae used to calculate the values, reference values and interpretation.

Dietary data were collected by using a 160 item Food Frequency Questionnaire (FFQ) to evaluate the patient’s dietary intake. It was adapted from a FFQ used in a South African study to include a greater variety of fruits, vegetables and phosphate containing foods and was evaluated for face and content validity [19]. It was administered by the dietitian researcher who interviewed participants. Food models and household metric measuring instruments such as spoons and cups were used to estimate portion sizes. Codes of the food items and the portion sizes were captured as daily intake. It was analyzed for the macronutrient and micronutrient composition per person per day, the SAFOODS database was used to analyze the data [20]. The mean/median dietary intake of nutrients were assessed and compared to the Kidney Disease Outcomes Quality Initiative (KDOQI) and Kidney Disease Improving Global Outcomes (KDIGO) recommendations [21,22,23].

Adjusted oedema free body weights were used to calculate the required nutrients where values were given per/kg of weight for energy and protein and as a percentage of energy for carbohydrate and fats, calculated in grams per day. Values or ranges for a specific nutrient were given as per the guidelines for cholesterol, fiber, vitamins and minerals. The nutrients were expressed as nutrient intake below, above and within recommendations as a percentage of participants.

Patient’s barriers to eating healthy were assessed with the responses being binary: the cost of food, time to cook or shop, motivation to cook or shop, lack of family support and the availability of shops to purchase food.

Data were tested for normality using various methods including Kolmogorov-Smirnoff, skewness and kurtosis values, histograms and Q-plots.

Basic descriptive tests were performed using frequencies and percentages for categorical data, means and standard deviations for normally distributed continuous data and medians and interquartile ranges for continuous data that was not distributed normally.

The following analytical tests were employed: correlations between protein intake and urea and creatinine, and between energy and urea and creatinine, as well as gender differences for anthropometry using t-tests and Chi-squared tests. Analysis of variance compared the following: BMI categories and dietary intake, financial income and dietary intake and between GFR and age, BMI, protein and energy intake. A *p* value of <0.05 was considered statistically significant.

## 3. Results

### 3.1. Socio-Demographics

Seventy participants entered the study. The mean age of the participants was 41.7 ± 11.8 years, with a slight predominance of females (53%). Most participants were employed, earning less than US $126 and had up to grade 11 schooling (Table 1).

### 3.2. Clinical

Hypertension was the most prevalent cause of renal failure and occurred in 35 (50%) of participants. Most participants had no oedema (44, 62.9%) and 31 (44.2%) had stage 5 CKD. The mean systolic blood pressure was 146.0 ± 25.5 mmHg and diastolic pressure was 81.0 ± 15.3 mmHg. (Table 2).

Fifty-one (73.9%) participants were receiving diuretics, 42 (60.9%) ACE inhibitors and 38 (55.1%) calcium channel blockers. Nearly a third of participants were on calcium and iron supplements. The majority of participants were on multiple medication combinations.

### 3.3. Anthropometry

Table 3 shows the mean weights which were significantly different (*p* = 0.03) for males and females. Similarly, the mean triceps were higher in females than males (*p* = 0.001).

The mean BMI was in the overweight category for the group with no gender differences. Regarding the BMI categories, 21 (30%) participants were overweight and 25 (36%) were obese. Obesity was more prevalent in females, with 46% of females being obese; whereas males were mainly in the normal weight category, with differences in gender being statistically significant (Chi^2^ = 8.9, *p* = 0.03) (Table 3). The mean MUAC for the group was in the obese category, with no gender differences.

The prevalence of underweight in this study was 4.3%, with only 3% of participants also having a wasted muscle and fat mass.

The majority of participants had a waist circumference in the high risk for chronic diseases category; this group comprised predominantly of females (57%), whereas males were in the normal waist category. The gender differences in waist circumference was significant (Chi^2^ = 8.0, *p* = 0.005). Thirty six (51.4%) of participants, had average arm muscle evenly spread between gender, and 40 (57.1%) also had average arm fat, with a significant gender differences between AFA categories (Chi^2^ = 12.2.0, *p* = 0.02).

### 3.4. Dietary Intake

Table 4 describes the mean/median intake of macronutrients and micronutrients and how they compare to recommended KDOQI ranges. The mean energy intake was 27 kcal per kg which is within the guidelines. The mean protein intake was higher than the guidelines at 1 g/kg, specifically animal protein at 64.8% of protein intake. Saturated fat intake was higher than guidelines at 10.7%. Added sugar and total sugar intake was high at 39 g and 70 g respectively. The mean intake of all other minerals and vitamins were within the recommendations, except for folate and vitamin D, which were lower than the guidelines.

Barriers such as cost of food were prevalent in 63% of patients, and 25% of patients lacked motivation to shop or cook.

**Figure 1:**
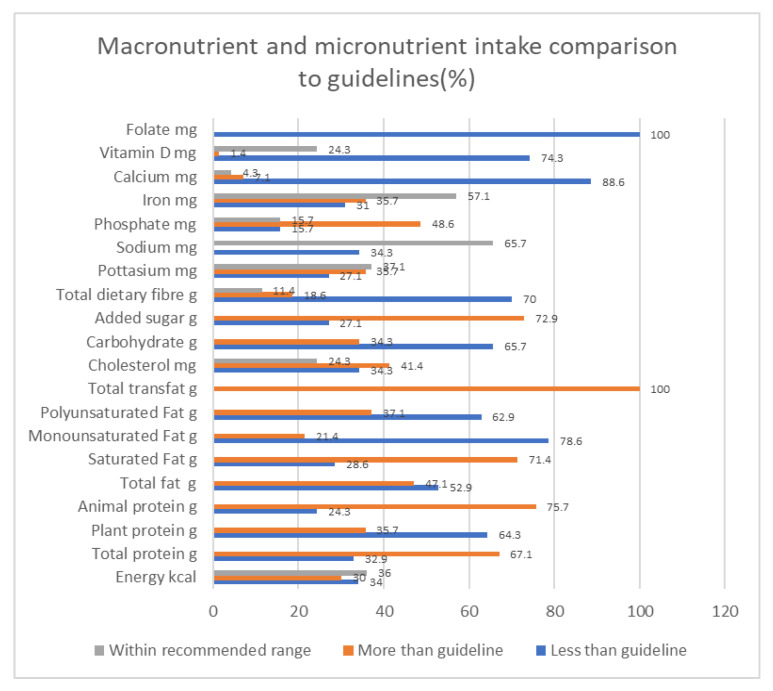
Nutrient intake compared to guidelines (% of participants).

We compared the intake of nutrients to dietary recommendations and grouped them according to the following criteria: within, more than or less than the recommended guidelines. (Figure 1). The following nutrients were consumed in quantities higher than the recommendations in more than 50% of participants: saturated fat, added sugar, trans fats, animal protein and total protein.

The following nutrients were consumed less than the recommendations in more than 50% of participants: vitamin D, calcium, fiber, folate, carbohydrates, total fat, polyunsaturated, monounsaturated fat and plant protein. Interestingly, of the 34% of participants with energy intakes less than recommendations, 16% were in the obese BMI category, with an average energy intake of only 14.3 ± 2.93 kcal/kg.

**Figure 2:**
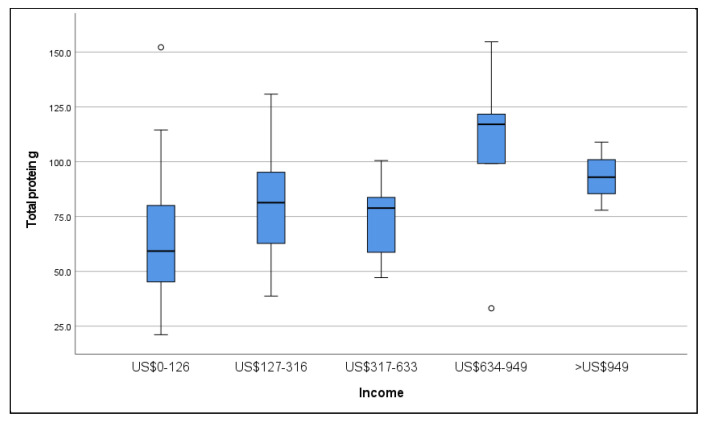
Boxplot of protein intake and socioeconomic status; ^○^ Indicates outliers.

A significant association was found between socioeconomic status and protein intake (*p* = 0.03), with the highest protein intake being in the second highest income (US $634–949) category (Figure 2). No other significant associations were found between socioeconomic status or BMI categories and any other macronutrient intakes.

### 3.5. Biochemistry 

The median urea and creatinine were raised and the GFR was low, which is consistent with end stage CKD. The median potassium, sodium and phosphate were in the normal ranges. The total cholesterol and low-density lipoprotein (LDL) were raised according to high risk cut off criteria. The median CRP was higher than the cut-off range and inflammation was present in 42 (60%) of participants. (Table 5) GFR categories (stage 3, 4 and 5) were compared to various variables, including age, BMI, waist, MUAC, protein and energy intake, with no significant differences found. Even though the BMI of those with stage 4 and 5 kidney failure were higher than those in Stage 3 kidney failure, the difference was not significant (*p* = 0.3). Similarly, waist circumference was also 8 cm higher in stage 4 than stage 3, also not statistically significant (*p* = 0.2). As expected, biochemical values were significantly higher for urea (*p* < 0.01), creatinine (*p <* 0.01), phosphate (*p <* 0.01) and potassium (*p* = 0.03) in stage 5 compared to stage 3 kidney failure due to the more advanced stage of the disease.

## 4. Discussion

The aim of this study was to assess the nutritional status of pre-dialysis CKD patients. We found a high prevalence overweight, obesity and abdominal obesity, low rates of undernutrition, and an unhealthy diet.

The mean age of the participants were 41.7 years, which is younger than most other CKD studies. In contrast to older studies [14,15], almost two thirds of participants were overweight or obese; 45% of females were predominantly obese. Abdominal obesity was present in 60% of participants, again predominantly in females. The BMI and waist circumference were higher in stage 4 and 5 CKD. Chan et al. [1], similarly to our study showed 62.4% of participants were overweight and obese, whereas Dierkes et al. [2] found an overweight and obesity prevalence of 65%. They also found a high rate of central obesity in 53% of patients. Epidemiological studies have also shown high prevalence of CKD in overweight and obese patients with higher BMIs in CKD stages 4–5 [5,16]. The high prevalence of overweight and obesity could relate to the high prevalence in the general population in South Africa in which 68% of women and 31% of men are overweight or obese [25]. The prevalence found in this study is similar to that of the background South African population in women, but there is a much higher prevalence of overweight and obesity in CKD men in this study. Most of these patients may have already been overweight and obese at diagnosis of CKD. This emphasizes the importance of weight control during the early stages of kidney disease to prevent further deterioration of the disease [3].

A majority of participants in the study had high arm muscle and high fat area which could relate to the increased BMI as other studies have shown [26,27]. Triceps and MUAC measurements were higher than reported [1,15,28] Muscle wasting was not found in obese participants in this study. Chan et al. [1], found a high rate of malnutrition including muscle wasting in their overweight and obese participants based on the SGA. The malnutrition was more significant as the age increased [1]. Dierkes et al. [2] also found a high rate of sarcopenia and sarcopenic obesity in their study, which also increased with age. Body composition changes associated with aging include increased fat mass and reduced muscle mass [2].

A possible explanation for the lack of muscle wasting and sarcopenia is this study is that the participants were much younger with an average age of 41 than the participants in both of the other studies, who averaged 65 years of age.

Hypertension was the most prevalent cause of CKD in participants as documented by the clinicians; this was similar to other studies and is also reflective of the hypertension rates in South Africa, where hypertension is present in 45% of men and 44% of women [13,14,25]. Overweight and obesity can account for 65–75% of the risk for hypertension [3]. Renal sinus fat have been associated with hypertension and the need for more hypertensive medications [3] The blood pressure was slightly higher in this study than recommended in CKD participants despite being on various combinations of anti-hypertensive medications. Hypertension together with other disorders linked to metabolic syndrome such as the obesity shown in this study can act synergistically to increase the risk for CKD and end stage kidney failure (ESKF). Sodium intake was lower than recommendations in a majority of participants. Sodium has been linked to hypertension and fluid retention.

Undernutrition in CKD has been a serious feature of CKD participants whereas obesity has received less attention due to the obesity paradox concept. Recent studies still show a high prevalence of undernutrition in some populations [1,14,15]. However, these studies use the criteria differently, leading to varying results. When the criteria used in the ISRNM were applied in our study, i.e., total cholesterol <100 mg, BMI <23 kg/m^2^ and dietary protein intake <0.6 g/kg, the prevalence of undernutrition was found to be zero. However, there were participants that were underweight as well as having wasted arm muscle mass and fat. The overall rate of undernutrition was therefore very low in this study and mainly confined to underweight participants with a wasted muscle and fat arm area which affected only 3% of participants. This is similar to that reported by Dierkes et al [2] who only found malnutrition in 3% using a nutritional risk score.

The dietary intake was indicative of unhealthy food choices, which is typical of a Western dietary food pattern. This study differs from others in terms of energy intake: 30% were higher than recommended range, while 32% were lower and 34% were in the normal range. Wlodarek et al. [29] reported that as many as 93% of their participants consume less energy than recommended. Steiber et al. [30] showed only 15% of participants met 75% of their requirements. Energy intake did not differ significantly amongst the BMI or GFR categories. A quarter of obese participants in this study were taking an average energy intake of 14 kcal/kg which may reflect underreporting. The latter is a common finding in obese patients [31]. The KDOQI energy requirement for obese participants are 303–305 kcal/kg [21]. More recent guidelines have advised 253–255 kcal/kg based on age, sex, activity level and body weight goals [23]. The lower energy recommendation of 25 kcal/kg should be recommended for overweight and obese subjects.

Protein intake was higher than recommended in 67% of participants and consisted mostly of animal protein. A protein intake of 0.60–0.8 g/kg has been recommended in CKD pre-dialysis participants; levels of 0.8 g/kg has been found to prevent a negative nitrogen balance together with a sufficient energy intake [32]. The most recent KDOQI guidelines suggest a protein intake of 0.550–0.6g/kg per day [23]. The high protein intake in most participants in this group differs from other studies, where intakes were reported to be mainly lower than recommended. A high protein intake is associated with a more rapid decline in kidney function and other complications [33]. This high intake of protein could also be reflective of the higher energy intake in some participants.

Protein intake did not differ in the GFR categories but was found to be significantly higher in participants with a higher income. Protein is often the most expensive food item when shopping, with processed protein sources being cheaper. The cost of food was found to be a barrier to purchasing healthy food options in this study. Although the average income was low, the animal protein intake was surprisingly high in this study. A low income increases the risk of disorders that predispose CKD progression and worsens outcomes in those who already have CKD [34]. Socio-economic factors are important to consider in CKD management. A majority of participants did not complete high school, a lower level of education has been associated with decreased adherence behavior in CKD due to lower health literacy [34].

The high animal protein intake could explain why dietary saturated fat intake and cholesterol intake were higher than recommended ranges, as well as why the total and LDL cholesterol levels were raised. Few participants were using statins. Although total fat is within the recommended ranges, the intake of healthier fats such as polyunsaturated and monounsaturated fat should be favored. These intakes were lower than recommendations for most participants.

Although the fiber intake was low for the most participants, the mean intake was good and marginally higher than reported in other studies [1,29]. Fiber intake is usually low in CKD participants due to dietary restrictions of wholegrains, fruit and vegetables. A high fiber intake has recently been found to reduce uraemic toxins and subsequently improve the gut microbiome in CKD [35]. Fiber regulates the bacteria in the gut and enhances the growth of saccharolytic bacteria [36]. These are essential as fuel cells for the colonic epithelial cells and regulatory T lymphocytes. These cells are already reduced in renal failure and this, together with a low fiber diet, may account for CKD-associated systemic inflammation [37]. Inflammation was high in this study, and it is usually associated with adverse outcomes such as increased mortality, progression of disease, increased cardiovascular disease, muscle wasting and cognitive decline [38].

Participants in this study had a very high sugar intake. Excessive intake of refined sugar can increase triglycerides and contribute to obesity. A recent review of refined sugars in CKD indicates that it is a driver of kidney disease and its consequences by causing metabolic derangements such as insulin resistance and uric acid production. This increases the conversion of glucose to fructose via the polyol pathway; this pathway has recently been implicated to cause kidney damage [39].

Phosphate intake was high in a larger percentage of participants, and calcium and vitamin D levels were low. Vitamin D intake is generally low in the general population and even more so in CKD participants [40]. A high phosphate intake was found in nearly half of the participants. High phosphate levels have been associated with increased cardiovascular disease due to vascular calcification as well as increased mortality [41]. Mineral and vitamin intake also varied. The low intake of folate and vitamin B6 intake are concerning since they are co-factors of homocysteine metabolism. Hyperhomocystenemia can contribute to cardiovascular disease, which is already a high risk in CKD participants [42]. Only a few participants were on a folate supplement and folate intake was lower than recommendations in all participants. Most participants had an adequate intake of other vitamins and minerals.

### Limitations of Study

There are various dietary intake assessment tools including 24-h recalls, food records and food frequency questionnaires, which are each subject to strengths and limitations. Although highly accurate data can be obtained with a food frequency questionnaire, measurement errors related to the methodology remain [43]. The relatively small sample size and strict exclusion criteria may have limited the inclusion of older participants. For muscle mass assessment, bio-electrical impedance measures and 24-h urine creatinine excretion may have given more accurate results, however this was not done due to resource limitations. Hypertension as a cause of CKD has been challenged, however we relied on the diagnosis that clinicians had recorded in the medical notes. Without renal biopsies being documented, it may be that hypertension was not the most prevalent cause of CKD.

## 5. Conclusions

This study set out to determine the nutritional status of CKD patients and found a variety of factors, both from a medical and social perspective predisposed this younger study population to CKD development and increased risk of cardiovascular disease. These factors include hypertension, inflammation, obesity, dietary, socioeconomic and education factors.

Obesity was highly prevalent, with a low prevalence of undernutrition. Dietary evaluation showed an unhealthy Western dietary pattern. The standard nutrition assessment methods gave a good overall impression of the nutritional status of patients.

Nutrition advice should also aim to improve the diet to a healthier pattern and target nutrition supplementation such as folate, calcium and vitamin D with more emphasis on obesity management such as stricter dietary recommendations, physical activity guidelines and behavior modification for weight loss. Medical and surgical options should be considered to treat more advanced obesity. A multi-faceted approach should be employed to deal with the disparities that predispose the population to the development of CKD as well as improving outcomes in those with CKD. On a population wide level, efforts should be employed to reduce the high prevalence of obesity, hypertension and other chronic conditions to prevent the development of CKD.

## Figures and Tables

**Table 1:** Sociodemographic profile of participants.

		*n*	Mean ± SD
Age (years)		70	41.7 ± 11.8
		*n*	Percent %
Gender	Male	33	47.1
	Female	37	52.9
Employment status	Full time	29	41.4
	Part time	5	7.1
	Unemployed	22	31.4
	Pensioner/Grant holder	4	5.7
	Other	10	14.2
Monthly Income	US $0–126	29	41.4
	US $127–316	18	25.7
	US $317–633	15	21.4
	US $634–949	5	7.1
	>US $949	3	4.3
Education level	Primary school	10	14.3
	Grade 8–11	32	45.7
	Grade 12	20	28.6
	University	1	1.4
	Technicon	7	10.0

**Table 2:** Clinical data of participants.

		*n*	Mean ± SD
Blood pressure (systolic) mmHg		64	146.0 ± 25.5
Blood pressure (diastolic) mmHg		64	81.0 ± 15.3
		*n*	%
Oedema	None	44	62.9
	Mild	15	21.4
	Moderate	8	11.4
	Severe	3	4.3
GFR stages	Stage 3	21	30.0
	Stage 4	18	25.7
	Stage 5	31	44.2
Cause Renal Failure	Polycystic kidney disease	6	8.6
	Hypertension	35	50.0
	Glomerular disease	13	18.6
	Other and unknown	16	22.9

GFR: Glomerular filtration rate.

**Table 3:** Anthropometry for total group and gender.

	Total Group *n* = 70	Male *n* = 33	Female *n* = 37	* *p* Value
	Mean ± SD			
Weight (kg)	76.8 ± 25.4	82.9 ± 23	71.4 ± 19.7	* 0.03
BMI (unit)	28.4 ± 7.0	28.4 ± 7.8	28.6 ± 6.4	* 0.90
Waist circumference (cm)	92.1 ± 16.8	94.9 ±19.5	91.6 ± 13.7	* 0.18
MUAC (cm)	31.0 ± 5.4	31.2 ± 5.1	30.5 ± 5.8	* 0.84
Triceps (mm)	21.0 ± 9.1	17.0 ± 9.0	24.0 ± 8.0	* 0.001
**BMI Categories**	*n* (%)			
Underweight	3 (4.3)	0	3 (8.1)	Chi^2^ = 8.9, *p* = ** 0.03
Normal weight	21 (30.0)	13 (39.4)	8 (21.7)	
Overweight	21 (30.0)	12 (36.4)	9 (24.3)	
Obese	25 (35.7)	8 (24.2)	17 (45.9)	
**Waist circumference Categories**				
Normal	28 (40)	18 (54.5)	10 (27.0)	Chi^2^ = 8.0, *p* = ** 0.005
Increased risk	13 (18.6)	7 (21.2)	6 (16.2)	
High risk	29 (41.4)	8 (24.2)	21 (56.8)	
**MUAC Categories**	*n* (%)			
Undernourished	5 (7.1)	0	5 (13.5)	Chi^2^ = 3.0, *p* = ** 0.22
Normal	17 (24.3)	9 (27.2)	8 (21.6)	
Overweight	9 (13.0)	5 (15.1)	4 (10.8)	
Obese	39 (55.7)	19 (57.5)	20 (54.0)	
**AMA Categories**	*n* (%)			
Wasted	1 (1.4)	1 (3.0)	0	Chi^2^ = 8.9, *p* = ** 0.06
Below average muscle	7 (10.0)	6 (18.2)	1 (2.7)	
Average muscle	36 (51.4)	17 (51.5)	19 (51.4)	
Above average muscle	13 (18.6)	5 (15.2)	8 (21.6)	
High muscle	12 (17.1)	3 (9.1)	9 (24.3)	
**AFA Categories**	*n* (%)			
Wasted	5 (7.1)	3 (9.1)	2 (5.4)	Chi^2^ = 12.2, *p* = ** 0.02
Below average fat	5 (7.1)	1 (3.0)	4 (10.8)	
Average fat	40 (57.1)	18 (54.5)	22 (59.5)	
Above average fat	10 (14.3)	2 (6.1)	8 (21.6)	
Excess fat	9 (12.9)	8 (24.2)	1 (2.7)	

* Independent *t*-tests. ** Chi-squared tests. BMI: body mass index; MUAC: mid upper arm circumference; AMA: arm muscle area; AFA: arm fat area.

**Table 4:** Recommended intakes versus actual intake of nutrients.

	Recommended Daily Allowances [21]	Actual Intake
		Mean ± SD
		*n* = 70
Energy kcal/kg	25–35 [23]	27
		2041.7 ± 732 kcal/kg
Total protein g/kg	0.6–0.8	1
	0.55–0.6 g/kg [23]	74.2 ± 28.4 g
Plant protein	50% of protein intake	34.2%
		25.4 ± 10.7 g
Animal protein	50% of protein intake	64.8%
		48.1 ± 21.2 g
Total fat	34% Energy	35.2%
		80.0 ± 34.9 g
Saturated Fat	<7% of Energy	10.7%
		24.3 ± 11.7 g
Monounsaturated Fat	<20% Energy	12.2%
		27.7 ± 14.4 g
Polyunsaturated Fat	<10% Energy	9.0% 20.6 ± 8.6 g
Total trans fat g	0	0.7 ± 0.5
Cholesterol mg	200–300	278.2 ± 133.7
Carbohydrate	55% Energy	49.3% E 251.9 ± 93.7 g
Added sugar g	25	39.1 (23.0, 59.1) *
Total sugars g	NA	69.9 ± 29.2
Total dietary fiber g	253–0	21.8 ± 9.7
Calcium mg	1000–1200	484.7 (349.0, 743.1) *
	800–1000 [23]	
Iron mg	101–8	13.0 ± 4.6
Phosphate mg	800–1000	1038.7 ± 420.6
Sodium mg	2400	2049 ± 965.1
	2300 [23]	
Potassium mg	2000–3000	2691.2 ± 932.7
Vitamin B6 mg	5	3.2 ± 1.3
Folate mg	1000	291.8 ± 118.0
Vitamin D mg	5–10	2.7 (1.8, 5.2) *

All values given as a mean SD, except as * median (interquartile range). Values calculated where g/kg of a nutrient is given or as range. Updated KDOQI guidelines were recently released: the reference [23] indicates where they differ, for the rest of the values, no specific values were provided in the update, it is therefore based on previous guidelines. NA, not applicable.

**Table 5:** Biochemistry profile of participants.

	Normal Ranges *	Actual Median and Interquartile Range
Urea mmol/L	2.1–7.1	16.3 (10.9, 25.3)
Creatinine umol/L	64–104	287.0 (183, 477.5)
GFR mL/min·1.73 m^2^	>60	19.0 (10.8, 31.2)
Potassium mmol/L	3.5–5.1	4.8 (4.3, 5.2)
Sodium mmol/L	136–141	142.0 (139, 144.0)
Phosphate mmol/L	0.78–1.42	1.4 (1.1, 1.5)
Total Chol mmol/L (high risk)	<4.5	4.9 (3.9, 5.7)
LDL (high risk) mmol/L	<2.6 **	2.7 (2.1, 3.3)
HDL mmol/L	>1.2	1.1 (1.0, 1.4)
TG mmol/L	<1.7	1.7 (1.2, 2.5)
CRP mg/L	<3 **	5.0 (1, 9)

* Normal ranges used by the South African National Health Laboratory (NHLS). ** Inflammation defined as a CRP > 3 mg/dL [13]. ** According to the American College of Cardiology CKD stage 3 and 4 is considered high risk for artherosclerotic cardiovascular disease [24]; GFR: glomerular filtration rate; Total Chol: Total cholesterol; LDL:Low-density lipoprotein, HDL: High density lipoprotein, TG: Triglycerides; CRP: C-reactive protein.

## Appendix A

**Table A1:** Anthropometric Measurements and Interpretations.

Measurement	Formula	Cut-Off Values	Interpretation
BMI [17]	Weight/height^2^	<18.49 kg/m^2^	underweight
		18.5–24.99 kg/m^2^	normal weight
		25–29.99 kg/m^2^	overweight
		>30 kg/m^2^	obese
Adjusted body weight [44]	aBW*_ef_*= BW*_ef_* + [(SBW − BW*_ef_*) × 0.25] ef: oedema free weight		
MUAC [45]		<23 cm females	Malnourished
		<22 cm males	Malnourished
		>28 females	Overweight
		>29 males	Overweight
		>30 females and males	Obese
WC [46]		<80 cm for females <94 cm for males	Normal
		between 80–88 cm for females between 94–102cm for males	Increased risk for disease
		>88 cm for females and >102 cm for males	High risk for disease
AFA/AMA area [44]	AFA = [MAC(cm) × TSF(cm)/2 π × TSF(cm)^2^]/4 π	<5th percentile	Wasted
	AMA = [MAC (cm) − (π × Triceps Skinfold Thickness (cm))]2/4 π		
		≥5th and ≤15th percentile	Below average muscle/fat
		≥15th and ≤85th	Average muscle/fat
		≥85th and ≤95th percentile	Above average muscle/fat
		>95th percentile	High muscle/fat

BMI: Body mass index; MUAC: Mid upper arm circumference; WC: Waist circumference; AFA: Arm fat area; AMA: Arm muscle area.
